# Advances in Neuromodulation for Chronic Pain

**DOI:** 10.3390/jcm11030874

**Published:** 2022-02-07

**Authors:** Maarten Moens, Lisa Goudman

**Affiliations:** 1 Department of Neurosurgery, Universitair Ziekenhuis Brussel, Laarbeeklaan 101, 1090 Brussels, Belgium; lisa.goudman@vub.be; 2 STIMULUS Consortium (reSearch and TeachIng neuroModULation Uz bruSsel), Vrije Universiteit Brussel, Laarbeeklaan 103, 1090 Brussels, Belgium; 3 Center for Neurosciences (C4N), Vrije Universiteit Brussel, Laarbeeklaan 103, 1090 Brussels, Belgium; 4 Pain in Motion (PAIN) Research Group, Department of Physiotherapy, Human Physiology and Anatomy, Faculty of Physical Education and Physiotherapy, Vrije Universiteit Brussel, Laarbeeklaan 103, 1090 Brussels, Belgium; 5 Department of Radiology, Universitair Ziekenhuis Brussel, Laarbeeklaan 101, 1090 Brussels, Belgium; 6 Research Foundation—Flanders (FWO), 1090 Brussels, Belgium

In the past decade, neuromodulation as a treatment option for pain took a huge interest in innovating and developing more effective paradigms to conquer chronic pain syndromes. Several device-developing companies introduced, in collaboration with clinical researchers and governmental authorities, randomized clinical and non-inferiority trials challenging their new paradigms over standard spinal cord stimulation [1,2,3,4,5,6].

These trials demonstrated impressive reductions in pain intensities by the new paradigms compared to the standard SCS, resulting in introducing terms such as “superiority” and “remitter”. Despite these monumental steps in the field of neuromodulation, predicting a good outcome for a single individual patient remains a challenge in daily practice. The challenge is not the know-how in introducing very specific statistical analyses in the world of neuromodulation but in defining what a good outcome means [7]. There are conflicting interests and definitions of success between patients, implanting physicians, companies, and authorities. For many years the primary outcome measurements in leading research were based on pain intensities and the amount of reducing painkillers. This oversimplification of a very complex syndrome, such as chronic pain, drove wedges between the different stakeholders. If we keep in mind that a personalized treatment for every chronic pain patient is the ultimate goal to reach for more independence for those patients, the definition of success by neuromodulation should be aligned to every stakeholder, including the patients. The first step towards the holy grail is to walk the extra mile for every patient, even for those who do not respond anymore to the initial paradigm. Salvage strategies and algorithms are gaining interest from researchers and clinicians [8,9,10,11,12,13]. Salvage therapy should not only consist of converting patients towards new paradigms but also introducing extra tools to regain freedom and independence in terms of patient empowerment. Within this evolution, the recognition that pain is much more than a biological problem is a mainstay. The social and professional dimensions of a chronic pain syndrome remain understudied. The other step is more an evolution in progress; several high-level studies immerse the original data in advanced statistical modelling and analyses. Coming from well-balanced and clinically relevant hypotheses, the next level of mathematical solutions is giving answers and predictions to clinicians in daily practice. The gap between the “sterile” clinical trials and real-world daily routine care should be bridged by clear interpretations, flowcharts, and prediction charts.

Thus, “advances in neuromodulation” are fights on different battlefields, resulting in a better life for chronic pain patients with a joint win for physicians, companies, and society.
